# Rapid molecular diagnostics of large deletional β^0^-thalassemia (3.5 kb and 45 kb) using colorimetric LAMP in various thalassemia genotypes

**DOI:** 10.1016/j.heliyon.2021.e08372

**Published:** 2021-11-12

**Authors:** Wanicha Tepakhan, Wittaya Jomoui

**Affiliations:** a Department of Pathology, Faculty of Medicine, Prince of Songkla University, Songkhla, Thailand; b Department of Pathology, Maha Chakri Sirindhorn Medical Center, Faculty of Medicine, Srinakharinwirot University, Nakhon Nayok, Thailand

**Keywords:** 3.5 kb deletion, 45 kb deletion, β-thalassemia, Loop-mediated isothermal amplification, Molecular testing

## Abstract

**Background:**

β-thalassemia is an inherited disorder that is reported worldwide. Two common β^0^-thalassemia mutations (3.5 kb and 45 kb deletions) are prevalent in Southeast Asia and Thailand. Identification of these defects is essential to population screening and prenatal diagnosis. We aimed to develop colorimetric LAMP based on a phenol red indicator and validate it on various thalassemia genotypes.

**Method:**

Colorimetric LAMP assays for detecting β^0^-thalassemia 3.5- and 45-kb deletions were developed and validated on 254 routine clinical samples. The results of the assays could be interpreted by the naked eye and compared with the gold standard gap-PCR.

**Results:**

A total of 254 samples related to seven phenotypes and 27 different genotype groups showed 100% concordance between the colorimetric LAMP assays and gap-PCR for detecting β^0^-thalassemia (3.5- and 45-kb deletions). The sensitivity, specificity, NPV, and PPV were calculated as 100% for both β^0^-thalassemia 3.5- and 45-kb deletion detection. The comparison of the usefulness of colorimetric LAMP assays and conventional methods was demonstrated in this study.

**Conclusions:**

The developed colorimetric LAMP assays are rapid, simple, and highly cost effective and can be interpreted by the naked eye. These assays should be applied for screening deletional β^0^-thalassemia in routine settings or small community hospitals in remote areas where thalassemia is highly heterogeneous.

## 1 Introduction

β-thalassemia, a defect in the β-globin gene that results in decreased production (β^+^-thalassemia) or absence (β^0^-thalassemia) of β-globin chain synthesis, is an inherited disorder commonly found in the Southeast Asian population [1]. Most mutations occur by point mutations, and some nucleotide deletions or insertions lead to frameshift mutations. However, large deletions are usually documented in the Thai population, especially in southern Thailand, where the heterogeneity of β-thalassemia mutations is greater than in other parts of the country [2, 3]. The frequency of the β-thalassemia trait throughout the country is approximately 3–9% [4, 5]. Furthermore, two common β^0^-thalassemia deletions, a 3.5-kb deletion (NC_000011.10:g.5224302-5227791del3490bp) and a 45-kb deletion (NG_000007.3:g.66258_184734del118477), have been reported with frequencies as high as 9.17% and 1.87%, respectively, among β-thalassemia carriers in southern Thailand [2]. The clinical manifestation of heterozygous β^0^-thalassemia is usually asymptomatic, but homozygous β^0^-thalassemia or compound heterozygous β^0^-thalassemia/hemoglobin (Hb) E can present with severe anemia and require regular blood transfusion. In Southeast Asia, a prevention and control program has been implemented to reduce the number of affected patients with these severe thalassemia diseases [2, 5, 6]. Thus, rapid and accurate molecular testing is the key to success. At present, molecular diagnosis of β-thalassemia with large deletion is performed by several polymerase chain reaction (PCR)-based techniques, such as gap-PCR, reverse dot blot (RDB) hybridization, and melt-curve analysis [2, 7, 8]. However, these methods require special instruments, personal skills, and post-PCR steps that take a long time, resulting in the limitation of applying these methods in a small community hospital or remote areas. Thus, it is necessary to develop a new method that is simple, low-cost, and has high sensitivity and specificity and rapid operation for the detection of β-thalassemia with large deletions.

Rapid molecular testing based on loop-mediated isothermal amplification (LAMP) is currently a favorable nucleic acid amplification method for molecular diagnostics. LAMP techniques are performed using *Bacillus stearothermophilus* (Bst) deoxyribonucleic acid (DNA) polymerase to amplify the reaction for 30–60 min under isothermal conditions [9]. The amplification results can be detected by several methods: visualizing the color shift of pH-sensitive dyes, fluorescent dyes under UV light, lateral flow, gel electrophoresis, or turbidity [10, 11, 12]. Of these, a colorimetric assay with a pH indicator (phenol red) system is designed to provide fast, precise visual detection of amplification based on the production of protons. The process occurs from the extensive LAMP reaction, producing a change in solution color from pink to yellow [10, 13]. This method is simple, rapid, and cost-effective, has high sensitivity and specificity and should be suitable for the detection of monogenetic disorders, i.e., thalassemia. In several studies, the LAMP method has been developed for the detection of both α-thalassemia with deletion mutations and β-thalassemia with point mutations [14, 15]. However, a technique for the detection of large deletional β^0^-thalassemia has not been reported or developed. In this study, we aimed to develop and evaluate a new technique for detecting two common deletional β^0^-thalassemia types, 3.5- and 45-kb deletions, based on a colorimetric LAMP assay in various thalassemia genotypes.

## 2 Materials and methods

### 2.1 Subjects and specimens

Ethical approval for this study was obtained from the Institutional Review Board of Srinakharinwirot University, Thailand (SWUEC/E-338/2563). Leftover DNA was recruited from our ongoing thalassemia screening program at the Department of Pathology, Maha Chakri Sirindhorn Medical Center, Faculty of Medicine, Srinakharinwirot University. Archival DNA samples were also obtained from a previous study (REC 62-073-5-2). A total of 254 samples with 27 genotypes were selected in the study. All samples were recorded with thalassemia screening (Mean corpuscular volume (MCV), Mean corpuscular hemoglobin (MCH), and Dichlorophenol-indophenol (DCIP) test), Hb analysis, and molecular diagnosis. Among these 254 samples, seven phenotypes were categorized: 142 samples had heterozygous β-thalassemia, 17 samples had heterozygous Hb E, 8 samples had homozygous Hb E, 16 samples had compound heterozygous β-thalassemia and Hb E, 36 samples had high Hb F determinants, two samples had β-thalassemia with high Hb F determinants, and 33 samples had a normal beta globin gene. All samples were blinded before being used to evaluate the developed method.

### 2.2 Thalassemia screening, Hb analysis and DNA analysis

All samples in this study were obtained from routine thalassemia services. All subjects who had screening results from automated blood cell counting (MCV and MCH) and DICP tests were routinely performed as previously described. The cutoffs of low MCV and MCH were 80 fL and 27 pg, respectively. Hb analysis was performed by capillary electrophoresis (Capillarys 2; Sebia, Lisses, France). A β-thalassemia heterozygous state was diagnosed using a cutoff HbA2 > 3.5% when the Hb type was A2A. Identification of β-thalassemia mutations is routinely performed in our laboratory using allele-specific PCR (AS-PCR), gap-PCR, and direct DNA sequencing analysis to detect point mutations, large deletions, and rare point mutations, respectively, as described elsewhere [2, 7, 16, 17].

### 2.3 Colorimetric LAMP assays

A novel rapid molecular detection method based on a colorimetric LAMP with phenol red assay was developed to detect large deletional β^0^-thalassemia (3.5- and 45-kb deletions) for the first time. All LAMP primer sets in this study were designed using Primer Explorer V5 software (http://primerexplorer.jp/lampv5e/index.html). The specific LAMP primer set containing the outer primers (F3 and B3) and the inner primers (FIP and BIP) for each β^0^-thalassemia (3.5-kb deletion) and β^0^-thalassemia (45-kb deletion) used in this study was protected according to petty patent submission number 2003002902. The strategy to design and select appropriated primers of LAMP technique with deletional β^0^-thalassemia (3.5- and 45-kb deletions), the primer set of F3, FIP should be located on 5′ breakpoint side whereas B3, BIP primer set located on 3'breakpoint side. Therefore, this is the key point for increasing the specific fragment of LAMP product. This colorimetric LAMP assay contains phenol red as a pH indicator, which changes the color from pink to yellow, and the results can be interpreted by the naked eye. Briefly, each colorimetric LAMP assay was performed in a 12.5-μL reaction mix containing 6.25 μL of WarmStart® Colorimetric LAMP 2x Master Mix (DNA & RNA); New England Biolabs (MA, USA), 0.25 μl of each 10 μM outer primer (F3 and B3), 0.5 μl of each 40 μM inner primer (FIP and BIP), 1 μl of 10–40 ng/μl genomic DNA, and the remainder distilled water. The LAMP mixture was incubated at an isothermal temperature of 65 °C for 65 min using a SimpliAmp thermal cycler (Thermo Fisher Scientific, Massachusetts, USA). Positive yellow color was observed by the naked eye compared with negative pink color.

### 2.4 Sensitivity and specificity of the colorimetric LAMP assay

**Figure 1:**
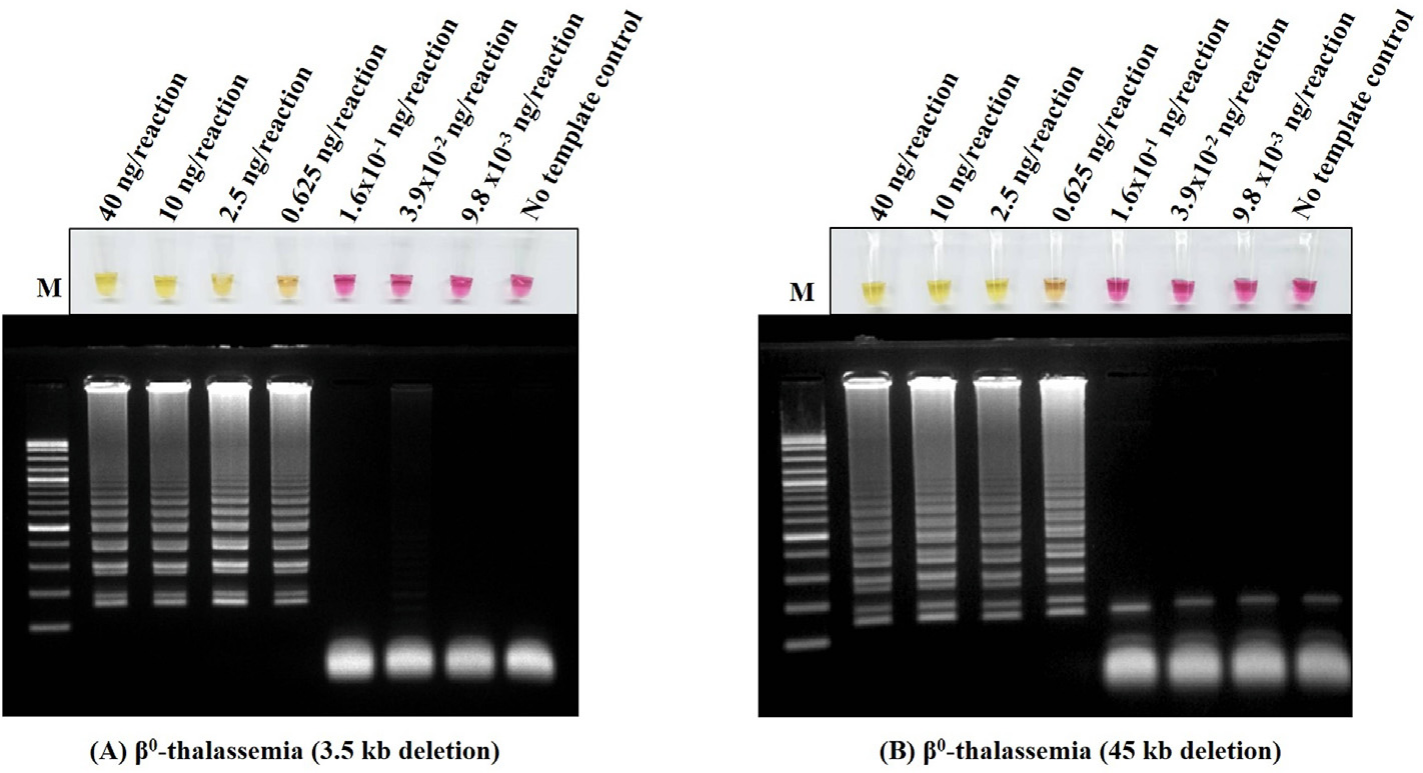
Determination of the lower limit of detection (LOD) of the developed colorimetric LAMP assays for detecting β^0^-thalassemia (3.5 kb and 45 kb deletion) and gel electrophoresis. The DNA four-fold serial dilutions were started with 40 ng/reaction to 9.8 × 10^−3^ ng/reaction, including (**A**) β^0^-thalassemia (3.5 kb deletion) (**B**) β^0^-thalassemia (45 kb deletion) assays.

**Figure 2:**
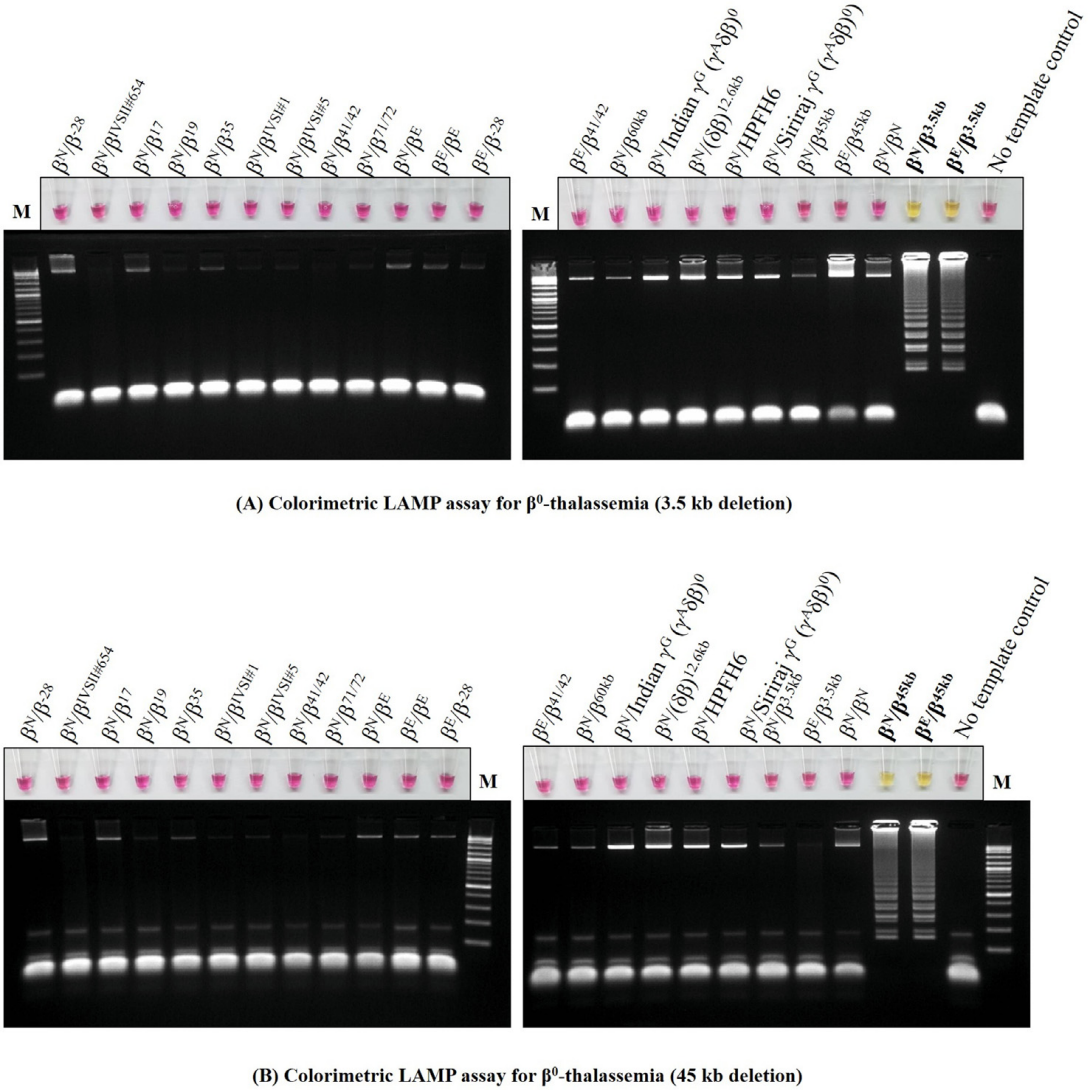
The Specificity assay of the colorimetric LAMP assays was demonstrated on samples with different 23 genotypes for detecting (**A**) β^0^-thalassemia 3.5 kb and (**B**) β^0^-thalassemia 45 kb deletion.

To determine the sensitivity assay, the lower limit of detection (LOD) of the colorimetric LAMP assay for the detection of both types of β^0^-thalassemia (3.5- and 45-kb deletions) was carried out using 4-fold serial dilution (DNA template range 40–9.8 × 10^−3^ ng/reaction). The results of colorimetric LAMP with serial dilution were observed and compared with gel electrophoresis, as shown in Figure 1. The specificity of the colorimetric LAMP assay to detect two large deletional β^0^-thalassemia types (3.5- and 45-kb deletions) was evaluated with 23 different genotypes, including (β^N^/β^−28^), (β^N^/β^IVSII#654^), (β^N^/β^17^), (β^N^/β^19^), (β^N^/β^35^), (β^N^/β^IVSI#1^), (β^N^/β^IVSI#5^), (β^N^/β^41/42^), (β^N^/β^71/72^), (β^N^/β^E^), (β^E^/β^E^), (β^E^/β^−28^), (β^E^/β^41/42^), (β^N^/β^60kb^), (β^N^/Indian γ^G^ (γ^A^δβ)^0^), (β^N^/(δβ)^12.6kb^), (β^N^/HPFH6), (β^N^/ Siriraj γ^G^ (γ^A^δβ)^0^)), (β^N^/β^45kb^), (β^E^/β^45kb^), (β^N^/β^3.5kb^), (β^E^/β^3.5kb^), and (β^N^/β^N^), which were compared with gel electrophoresis, as shown in Figure 2.

### 2.5 Detection of large deletional β^0^-thalassemia (3.5- and 45-kb deletions) using the colorimetric LAMP assay in various thalassemia genotypes

A total of 254 leftover DNA samples with several thalassemia genotypes were examined in blinded trials with the colorimetric LAMP assay as described. The results are summarized in Table 1 and were compared between the colorimetric LAMP assay and thalassemia genotypes based on conventional gap-PCR as the gold standard for detecting large deletional β^0^-thalassemia (3.5- and 45-kb deletions) [7, 16]. Furthermore, the sensitivity, specificity, NPV, and PPV of the colorimetric LAMP assay to detect large deletional β^0^-thalassemia (3.5- and 45-kb deletions) in clinical samples were calculated as shown in Table 2 & Table 3.

**Table 1:** A total of 254 unrelated clinical samples with thalassemia screening (MCV, MCH, DCIP), Hb analysis, β-globin gene genotype, and colorimetric LAMP assays for detecting β^0^-thalssemia (3.5 kb and 45 kb deletion). P & N are positive and negative, respectively. The values indicated as mean with standard deviation or as raw data where appropriate.

β-thalassemia phenotype	β-globin gene	N (254)	Thalassemia screening			Hb analysis			Colorimetric LAMP for β^0^-thalssemia	
			MCV (fL)	MCH (pg)	DCIP	Hb Types	%Hb A2/E	%Hb F	3.5 kb	45 kb
Normal	β^N^/β^N^	33	84.2 ± 9.5	28.8 ± 3.5	N	A2A	2.7 ± 0.9	0.0	N	N
Heterozygous β-thalassemia	β^N^/β^−28^	2	68.5, 74.3	22.1, 26.8	N	A2A	5.8, 5.6	0, 2.1	N	N
	β^N^/β^8/9^	1	59.4	19.3	N	A2A	5.2	0.3	N	N
	β^N^/β^17^	14	58.4 ± 5.3	18.8 ± 2.8	N	A2A	5.3 ± 0.6	2.4 ± 2.4	N	N
	β^N^/β^19^	4	72.1 ± 6.0	23.6 ± 1.5	N	A2A	4.4 ± 0.1	0.7 ± 0.4	N	N
	β^N^/β ^IVSI#1^	5	65.9 ± 3.6	21.0 ± 1.5	N	A2A	5.1 ± 0.3	2.8 ± 1.8	N	N
	β^N^/β ^IVSI#5^	2	66.4, 55.6	21.8, 17.5	N	A2A	5.3, 4.6	1.7, 0	N	N
	β^N^/β^35^	1	70.4	23.4	N	A2A	4.4	0.4	N	N
	β^N^/β^41/42^	21	64.5 ± 4.1	20.9 ± 1.8	N	A2A	5.4 ± 0.4	2.1 ± 1.8	N	N
	β^N^/β^71/72^	1	61.9	19	N	A2A	5.5	0.0	N	N
	β^N^/β ^IVSII#654^	4	60.2 ± 3.0	19.8 ± 0.8	N	A2A	5.3 ± 0.3	1.2 ± 1.8	N	N
	β^N^/β^3.5kb^	68	65.3 ± 4.3	21.1 ± 1.7	N	A2A, A2FA	6.9 ± 0.7	6.8 ± 3.0	**P**	N
	β^N^/β^45kb^	18	64.5 ± 4.1	20.9 ± 1.8	N	A2A, A2FA	6.5 ± 0.5	3.6 ± 1.9	N	**P**
	β^N^/β^60kb^	1	68.3	23.1	N	A2FA	5.3	23.9	N	N
Heterozygous Hb E	β^N^/β^E^	17	63.0 ± 9.2	20.8 ± 3.6	P	EA	24.2 ± 5.9	2.1 ± 1.2	N	N
Homozygous Hb E	β^E^/β^E^	8	55.3 ± 4.2	18.8 ± 2.0	P	EE, EE/EF	94.4 ± 6.7	6.4 ± 6.8	N	N
Compound heterozygous β-thalassemia/Hb E	β^E^/β^−28^	2	60.7, 50.8	20.5, 16.3	P	EFA	55.7, 61.4	20.1, 15.3	N	N
	β^E^/β^17^	1	67.4	20.0	P	EF	50.0	39.0	N	N
	β^E^/β^41/42^	4	53.3 ± 5.6	17.7 ± 1.4	P	EF	58.4 ± 20.3	41.5 ± 20.2	N	N
	β^E^/β^3.5kb^	6	62.9 ± 4.2	20.5 ± 1.5	P	EF	32.1 ± 25.1	48.9 ± 6.0	**P**	N
	β^E^/β^45kb^	3	71.5 ± 7.8	21.0 ± 1.8	P	EF	43.6 ± 12.5	39.5 ± 10.7	N	**P**
High HbF determinants	β^N^/(δβ)^12.5kb^	12	70.36 ± 4.2	22.5 ± 2.3	N	A2FA	2.3 ± 0.3	19.4 ± 5.6	N	N
	β^N^/HPFH6	8	81.8 ± 5.0	27.5 ± 1.5	N	A2FA	2.1 ± 0.2	25.8 ± 1.7	N	N
	β^N^/Siriraj γ^G^ (γ^A^δβ)^0^	9	76.4 ± 5.2	25.6 ± 1.5	N	A2FA	2.1 ± 0.1	23.6 ± 1.0	N	N
	β^N^/Indian γ^G^ (γ^A^δβ)^0^	7	74.5 ± 3.3	23.7 ± 0.7	N	A2FA	2.5 ± 0.3	18.5 ± 2.5	N	N
β-thalassemia with high HbF determinants	β^45kb^/(δβ)^12.5kb^	1	68.0	22.7	N	A2F	2.0	98	N	**P**
	β^45kb^/HPFH6	1	77.0	21.8	N	A2F	1.9	98.1	N	**P**

**Table 2:** The sensitivity, specificity, positive and negative predictive values of colorimetric LAMP assays compared with gap-PCR analysis as a gold standard for detecting β^0^-thalssemia (3.5 kb deletion) in various thalassemia genotypes.

Colorimetric LAMP	Gap-PCR analysis		Total
	Positive	Negative	
Positive	74	0	74
Negative	0	180	180
Total	74	180	254

Sensitivity	=	(74/74) x 100	=	100	%
Specificity	=	(180/180) x 100	=	100	%
Positive predictive value	=	(74/74) x 100	=	100	%
Negative predictive value	=	(180/180) x 100	=	100	%

## 3 Results

In Table 1, the thalassemia screening, Hb analysis, and β-globin genotyping results among these 254 samples are shown. A total of 33 wild-type sample with negative screening (MCV, MCH, and DCIP) was Hb type as A₂A (HbA₂ <3.5%). In the second group, all 142 samples related to heterozygous β-thalassemia had low MCV and MCH and were DCIP negative. Hb analysis of these subjects revealed HbA2 > 3.5% with A2A or A2FA type. The heterozygous β-thalassemia group had 13 different genotypes, including (β^N^/β^−28^; n = 2) (β^N^/β^8/9^; n = 1), (β^N^/β^17^; n = 14), (β^N^/β^19^; n = 4), (β^N^/β^IVSI#1^; n = 5), (β^N^/β^IVSI#5^; n = 2), (β^N^/β^35^; n = 1), (β^N^/β^41/42^; n = 21), (β^N^/β^71/72^; n = 1), (β^N^/β^IVSII#654^; n = 4), (β^N^/β^3.5kb^; n = 68), (β^N^/β^45kb^; n = 18), and (β^N^/β^60kb^; n = 1). In the third group, 17 samples with heterozygous Hb E (Hb type: EA; Hb E levels of 24.2 ± 5.9) had variable MCV and MCH, whereas DCIP was positive. The fourth group with low MCV and MCH was DCIP positive and had Hb type EE or EE/EF, revealing homozygous E in all eight cases, with Hb E levels of 94.4 ± 6.7. In the fifth group, 16 samples with low MCV and MCH were DCIP positive and had compound heterozygous β-thalassemia and Hb E (Hb type: EFA, or EF). Among these 16 samples, five different genotypes were represented: (β^E^/β^−28^; n = 2), (β^E^/β^17^; n = 1), (β^E^/β^41/42^; n = 4), (β^E^/β^3.5kb^; n = 6), and (β^E^/β^45kb^; n = 3). Next, the high HbF determinant group was associated with high and low of MCV and MCH, and were DCIP negative in thalassemia screening. These groups were Hb-type A2FA, including four common types: (β^N^/(δβ)^12.6kb^; n = 12), (β^N^/HPFH6; n = 8), (β^N^/Siriraj γ^G^ (γ^A^δβ)^0^); n = 9), and (β^N^/Indian γ^G^ (γ^A^δβ)^0^; n = 7). In the last group, two samples with (β^45kb^/(δβ)^12.6kb^) and (β^45kb^/HPFH6) had low MCV and MCH and were DCIP negative, whereas the Hb type was A2F, while Hb A was not found.

To assess the sensitivity of the developed colorimetric LAMP assays, LOD values were determined, as shown in Figure 1. The concentration of DNA template was diluted with 4-fold serial dilutions ranging from 40 to 9.8 × 10^−3^ ng/reaction. The LOD of colorimetric LAMP assays was 0.625 ng/reaction in both β^0^-thalassemia 3.5-kb (Figure 1A) and 45-kb deletion detection (Figure 1B). The analytical specificity tests of colorimetric LAMP assays for the detection of β^0^-thalassemia 3.5- and 45-kb deletions indicated no cross-reactivity with 21 other β-globin gene defects. We demonstrated the results of specificity assays comparing naked eye colorimetric assays and gel electrophoresis in β^0^-thalassemia 3.5-kb (Figure 2A) and 45-kb deletion detection (Figure 2B). The samples for the cross-reactivity test were selected with different types of β-globin gene defects, including point mutations, frameshift mutations, and deletions.

Application to clinical samples was performed on 254 samples with 27 genotypes. The results of colorimetric LAMP assays to detect β^0^-thalassemia 3.5- and 45-kb deletions are summarized in Table 1. These samples included seven phenotypic and 27 genotypic groups, as listed in the table. Among these 254 samples, 74 carried β^0^-thalassemia (3.5-kb deletion), including 68 heterozygous β^0^-thalassemia (3.5-kb deletion) and 6 compound heterozygous β^0^-thalassemia (3.5-kb deletion)/HbE. Twenty-three samples with β^0^-thalassemia (45-kb deletion) related to four genotypes (β^N^/β^45kb^; n = 18), (β^E^/β^45kb^; n =3), (β^45kb^/(δβ)^12.6kb^), and (β^45kb^/HPFH6) were included in the study. The remaining 157 samples had 21 different genotypes, as summarized in Table 1. All 74 samples with β^0^-thalassemia (3.5-kb deletion) tested positive in colorimetric LAMP assays for the detection of β^0^-thalassemia 3.5-kb deletion, while the remaining 180 samples with no β^0^-thalassemia (3.5-kb deletion) were negative in the same assay. Because of the 100% concordance result between colorimetric LAMP assays and gap-PCR as the gold standard method for the detection of β^0^-thalassemia 3.5-kb deletion, the sensitivity, specificity, NPV, and PPV in clinical diagnosis were calculated as 100% in all parameters, as shown in Table 2.

The colorimetric LAMP assays for the detection of β^0^-thalassemia 45-kb deletion were positive in all 23 samples with β^0^-thalassemia (45-kb deletion), while the remaining 231 samples with 23 different genotypes tested negative in both the colorimetric LAMP assays and conventional gap-PCR for detection of β^0^-thalassemia 45-kb deletion. Thus, the sensitivity, specificity, PPV, and NPV of the colorimetric LAMP assays for β^0^-thalassemia 45-kb deletion detection were 100% for all parameters. These statistics were calculated as shown in Table 3. No false-positives or false-negatives were observed in either colorimetric LAMP assay for β^0^-thalassemia 3.5- or 45-kb deletion detection.

**Table 3:** The sensitivity, specificity, positive and negative predictive values of colorimetric LAMP assays compared with gap-PCR analysis as a gold standard for detecting β^0^-thalssemia (45 kb deletion) in various thalassemia genotypes.

Colorimetric LAMP	Gap-PCR analysis		Total
	Positive	Negative	
Positive	23	0	23
Negative	0	231	231
Total	23	231	254

Sensitivity	=	(23/23) x 100	=	100	%
Specificity	=	(231/231) x 100	=	100	%
Positive predictive value	=	(23/23) x 100	=	100	%
Negative predictive value	=	(231/231) x 100	=	100	%

## 4 Discussion

More than 200 β-thalassemia mutations have been reported worldwide, and each population has its own spectrum. In Thailand, more than 30 β-thalassemia mutations have been reported and have different spectra in each region. In Southeast Asia and Thailand, severe β-thalassemia disease is associated with homozygous β^0^-thalassemia or compound heterozygous β^0^-thalassemia/Hb E [7, 18]. Two large deletional β^0^-thalassemia events, including a 3.5-kb deletion and a 45-kb deletion, are prevalent in Thailand, especially in the southern part of the country [2, 3]. These deletions are targeted to prevention and control in a fetus at risk of severe disease [2]. According to Table 1, Hb analysis showed that the expression levels of HbA2 and HbF in heterozygous large deletional β^0^-thalassemia were higher than those in other β^0^-thalassemia mutations. The high expression of HbA2 and HbF in both types of large deletional β^0^-thalassemia could be described based on the deletion, which shifts the enhancer to nearly its globin genes [19]. The investigation of 3.5-kb and 45-kb deletion in samples with high Hb A₂ (more than 6%) should be done first, and followed by AS-PCR or sequencing for other mutations.

As shown in Figures 1&2, we demonstrated that our colorimetric LAMP assays to detect β^0^-thalassemia (3.5- and 45-kb deletions) are simple and have high sensitivity and specificity in the developed assays. The LODs of colorimetric LAMP assays were 0.625 ng/reaction in both assays, which ensured that these two assays could be suitable for use with general DNA sample types. In practice, the concentration of DNA extracted from clinical specimens is usually more than 10 ng/μL and is derived using several methods [20]. In this study, the concentration of DNA samples range from 10 to 45 ng/uL. To ensure that our assays had no cross-reaction with other β-thalassemias, high HbF determinant groups, or wild-type, we demonstrated the specificity of the LAMP primers and condition assays, as shown in Figure 2. Among 21 other genotypes that did not carry a β-thalassemia 3.5- or 45-kb deletion depending on each assay, negative results with a pink color of phenol red were clearly observed by the naked eye. Conversely, samples carrying a large deletional β-thalassemia 3.5-kb deletion (Figure 2A) or 45-kb deletion (Figure 2B) were strongly positive and yellow. Colorimetric LAMP assays are mostly developed for the molecular detection of pathogens, i.e., viruses and bacteria, while their use in the detection of genetic diseases is less common [10, 13, 21, 22]. Because each pathogen has a specific nucleotide sequence, LAMP primers could be easily designed, whereas most human genetic diseases are caused by point mutations or single-nucleotide polymorphisms, which are difficult to distinguish in LAMP assays. Thus, several studies associated with LAMP assays are documented in microbiology.

To evaluate the developed colorimetric LAMP assays in clinical samples, we evaluated 254 samples with 27 genotypes that cover common β-globin gene defects in this region. As shown in Table 1, the results of the developed colorimetric LAMP assays showed 100% concordance with conventional gap-PCR to detect β-thalassemia (3.5- and 45-kb deletions). The sensitivity, specificity, NPV, and PPV were all 100% in both assays, as shown in Table 2 & Table 3. These results indicate that the developed colorimetric LAMP assays are highly effective compared to conventional gap-PCR. However, the major shortcomings of LAMP based on colorimetric should be aware because it is heavy reliance on indirect detection methods like non-specific dyes, which may be leads to the detection of false positive results. In this study, we found that both target sequences were successfully amplified because the LAMP primer set was designed by overlapping breakpoints of the deletions. Thus, these re-jointed nucleotide sequences of the deletions are less similar to other alignments of the β-globin gene. Previous studies were documented on colorimetric LAMP assays for detecting five β-thalassemia genes, including 654M, 41/42M, −28M, 17M, and 27/28M. These β-thalassemia results in false-positives and false-negatives in 41/42M, 17M, and 654M individuals, which may result from the mutation type and not large deletion types. However, previous study was successfully developed for detecting β-thalassemia −28M and 27/28M with 100% concordance [15]. Several studies have shown more false-positive results, possibly because most genetic defects were closely related to the wild-type gene sequence before mutation, which is easy to false-amplify [15, 23, 24]. Thus, the LAMP assay should be initiated for screening genetic diseases rather than diagnostic diseases.

Due to the utility of the novel developed assay for detecting β-thalassemia (3.5- and 45-kb deletions), we compared the novel assays with other molecular tests for these defects, as shown in Table 4. DNA analysis for detecting β-thalassemia (3.5- and 45-kb deletions) has been reported based on gap-PCR because these defects are related to deletion type. Several techniques, including gel electrophoresis, melting curve analysis, and reverse dot blot, have been developed based on gap-PCR, which is performed using thermal cycler machines and requires post-PCR steps [2, 7, 8]. Furthermore, the high cost of thermal cycler machines, especially those capable of real-time PCR, limits their use in remote areas or in small community hospitals. In contrast, LAMP assays can be conducted on isothermal and inexpensive instruments like dry bath. Unfortunately, in this study, we developed the LAMP assay based on isothermal with PCR machine not dry bath. For technical practice, LAMP assays have many advantages, such as their high sensitivity and high specificity, and they are simple and fast procedures. Furthermore, the time consumed in the gap-PCR-based method could be more than 2 h (2–7 h, depending on the assay), while LAMP assays take less than 1.30 h. In addition, we also demonstrated the cost effectiveness in our setting using different methods. The costs per specimen of a gap-PCR assay, including gel electrophoresis, melting curve analysis, and RDB, would be USD16, USD 5, and USD17, respectively [8]. Conversely, the cost of the developed colorimetric LAMP assays would be USD 6 per specimen.

**Table 4:** The comparison of time consumed, cost, and requiring instrument for detecting β^0^-thalssemia (3.5 and 45 kb deletion) in three conventional gap-PCR based and the developed colorimetric LAMP assays in our setting.

Method of detection	Time (minute)	Cost/sample (USD)	Instrument
Gap-PCR with gel electrophoresis	240	16	PCR machine Gel electrophoresis and imaging Systems
Gap-PCR with melting curve analysis	120	5	Real-time PCR machine
Gap-PCR with reverse dot blot (RDB)	420	17	PCR machine
Colorimetric LAMP assays	90	6	Isothermal equipment (dry bath)

## 5 Conclusion

In conclusion, the developed colorimetric LAMP assays are rapid, simple, and highly cost effective, do not require a post-PCR step, and can be interpreted by the naked eye. These colorimetric LAMP assays should be applied in screening for large deletional β^0^-thalassemias (3.5 kb and 45 kb) in routine settings where heterogeneity of β-thalassemia is common. Finally, these assays should be established, especially at a small community hospitals in remote areas.

## Ethical approval

Ethical approval for this study was obtained from the Institutional Review Board of Srinakharinwirot University, Thailand (SWUEC/E-338/2563).

## Declarations

### Author contribution statement

Wanicha Tepakhan: Conceived and designed the experiments; Analyzed and interpreted the data; Contributed reagents, materials, analysis tools or data; Wrote the paper.

Wittaya Jomoui: Conceived and designed the experiments; Performed the experiments; Analyzed and interpreted the data; Contributed reagents, materials, analysis tools or data; Wrote the paper.

### Funding statement

This work was supported by a research grant from HRH Princess Mahachakri Sirindhorn Medical Center, Faculty of Medicine, Srinakharinwirot University (Contract No 100/2564).

### Data availability statement

Data included in article/supplementary material/referenced in article.

### Declaration of interests statement

The authors declare no conflict of interest.

### Additional information

No additional information is available for this paper.
